# Case Report: Multidrug-Resistant Tuberculosis and COVID-19 Coinfection in Port-au-Prince, Haiti

**DOI:** 10.4269/ajtmh.20-0851

**Published:** 2020-09-25

**Authors:** Stalz Charles Vilbrun, Laurent Mathurin, Jean W. Pape, Daniel Fitzgerald, Kathleen F. Walsh

**Affiliations:** 1 The Haitian Group for the Study of Kaposi’s Sarcoma and Opportunistic Infections (GHESKIO), Port-au-Prince, Haiti;; 2 Weill Cornell Medicine, Center for Global Health, New York, New York

## Abstract

The COVID-19 pandemic poses a unique threat to patients with multidrug-resistant tuberculosis (MDR-TB). We describe a case of a patient with pulmonary MDR-TB and COVID-19 in Port-au-Prince, Haiti, and highlight the challenges and approach to managing a patient with both diseases.

## INTRODUCTION

COVID-19 is surging globally, threatening to overwhelm health systems and adversely impact the care of other diseases. Because underlying lung disease is associated with higher mortality in patients with COVID-19,^1^ patients with tuberculosis (TB) who become ill with COVID-19 may have increased risk of poor outcomes. This is especially true for patients with multidrug-resistant TB (MDR-TB) because they have an increased number of cavities and lobar volume decrease compared with patients with drug-susceptible TB.^2^ We present a case of a patient with MDR-TB and COVID-19 admitted to our MDR-TB hospital in Port-au-Prince, Haiti, and highlight the key screening, management, and infection control challenges faced by these diseases.

## CASE DESCRIPTION

**Figure 1.**
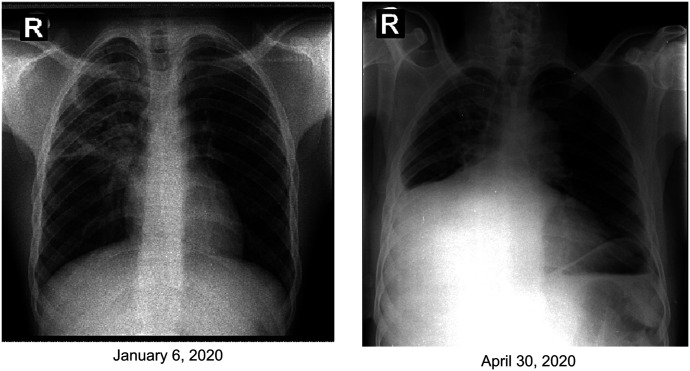
Chest radiograph at first presentation and when coinfected with SARS-CoV-2.

On January 6, 2020, a 26-year-old HIV-negative Haitian man presented to The Haitian Group for the Study of Kaposi’s Sarcoma and Opportunistic Infections (GHESKIO) Center in Port-au-Prince, Haiti, with a history of chronic cough, fever, and weight loss. He was evaluated by the medical staff with a physical examination, chest radiograph, and sputum analysis. The chest radiograph (Figure 1) demonstrated a large right upper lobe cavity, with right middle lobe opacity and right hilar fullness. The GeneXpert (Cepheid, Sunnydale, CA) sputum test was positive for the presence of *Mycobacterium tuberculosis* with probable resistance to rifampin. On January 9, during his follow-up visit, the patient presented to GHESKIO’s inpatient MDR-TB hospital, one of two main treatment facilities for MDR-TB in Haiti. Before hospitalization and initiation of therapy, the patient left against medical advice and was lost to follow-up. Subsequent liquid and agar culture-based drug susceptibility testing performed on the patient’s diagnostic sputum sample indicated high level of isoniazid resistance, rifampin resistance, and susceptibility to all other antituberculous agents.

On April 30, this patient returned to GHESKIO with persistent fever and cough, an additional 11-kg weight loss, and new onset dyspnea at rest. He was admitted to the GHESKIO MDR-TB hospital in a single room and isolated from other patients. Because of his rapid decline and in accordance with GHESKIO infection control policy, he was tested for COVID-19, and only designated medical staff with personal protective equipment appropriate for droplet and airborne precautions provided care. He underwent medical evaluation including repeat chest radiograph which was nonspecific for COVID-19 (Figure 1) and routine blood testing. Vital signs were notable for tachycardia with a heart rate of 130 beats/minute and an oxygen saturation of 93% on room air. On physical examination, he was noted to be cachectic, with decreased breath sounds in the right middle and upper lung, and with bilateral lower extremity edema. Complete blood count demonstrated a white blood cell count of 12,100 with 13% lymphocytes (expected range, 24–58%) and a hemoglobin level of 9,600 g/dL. Metabolic panel demonstrated a creatinine level of 0.4 mg/dL, a blood urea nitrogen of 7 mg/dL, a glucose content of 154 mg/dL, and an aspartate aminotransferase level of 110 U/L (normal range, 14–59). An all-oral daily antituberculous regimen composed of bedaquiline 400 mg, levofloxacin 1,000 mg, linezolid 600 mg, clofazimine 100 mg, and pyrazinimide 1,600 mg was initiated.

The patient’s initial COVID-19 test (Radi COVID-19, KH Medical, Hanam-si, Korea) resulted “indeterminate” and was repeated the following day. On May 2, 2 days following admission, the patient’s COVID-19 test resulted positive, and the patient was transferred from the main MDR-TB hospital to a separate building to prevent spread of the SARS-CoV-2 virus to other hospitalized patients. During hospitalization, the patient did not require oxygen therapy. Three weeks following diagnosis, the patient had recovered from COVID-19, and repeat SARS-CoV-19 PCR test was negative. He continued on antiTB therapy without complication.

## DISCUSSION

COVID-19 and MDR-TB present significant challenges in screening, management, and infection control. To the best of our knowledge, this is the first case report of a patient with MDR-TB and COVID-19 in a resource-limited setting. We provide recommendations for overcoming the challenges inherent in managing these two diseases (Table 1).

**Table 1:** Recommendations for managing MDR-TB and COVID-19 coinfection

Screening
Develop specialized triage protocols to identify and screen patients suspected for COVID-19 who are at high risk of MDR-TB, such as known MDR-TB contacts or TB patients with sudden clinical deterioration
Identify COVID-19 testing platforms that complement existing laboratory systems
Natural history and treatment
Frequently monitor MDR-TB patients for symptoms of COVID-19 that may interfere with their ability to take MDR-TB medications
Be especially cautious of MDR-TB patients with significant structural lung damage because they may be at high risk for poor outcomes with COVID-19
Infection control
Implement strict screening for COVID-19 among all MDR-TB patients who require hospitalization
Transition stable MDR-TB patients on all-oral regimens to outpatient care

MDR-TB = multidrug-resistant tuberculosis.

### Screening.

Multidrug-resistant TB requires laboratory testing to identify rifampin resistance and cannot be diagnosed solely on clinical history. This is challenging at a time when laboratories are overwhelmed with SARS-CoV-2 testing.^3^ To efficiently screen for both diseases, clinics should implement specialized triage protocols to prioritize those patients at highest risk for MDR-TB. Patients suspected of having COVID-19 but with insidious symptom onset, acute deterioration in the setting of chronic symptoms, known MDR-TB contacts, or cavitary disease on chest radiograph should be prioritized for MDR-TB and COVID-19 testing. Because 20% of new MDR-TB cases occur in those previously treated for TB,^4^ any patient with known TB presenting with symptom recurrence should be tested for MDR-TB and COVID-19. We implemented specialized triage protocols for MDR-TB screening at GHESKIO to prioritize those at risk for both diseases; similar protocols have been developed in other TB-endemic countries.^5^

Second, healthcare facilities in resource-poor countries should use compatible diagnostic technology to conserve as many resources as possible. The WHO has stated GeneXpert (Cepheid) is highly accurate as an initial screening test for MDR-TB.^6^ Cepheid also created a test to detect SARS-CoV-2 which uses the same equipment as its TB tests.^7^ Many clinics in resource-poor countries already have the Cepheid TB platform and could leverage this to provide COVID-19 testing without incurring the additional costs of new equipment. Unfortunately, Cepheid COVID-19 testing kits have been preferentially distributed in high-income countries. This disparity must be addressed so that resource-poor countries can afford diagnostic testing for COVID-19.

### Treatment.

COVID-19 poses a serious threat to successful anti-MDR-TB drug therapy which requires five medications during the intensive phase. Nineteen percent of COVID-19 patients experience nausea and vomiting which may prevent adherence to MDR-TB drugs.^8^ The WHO strongly encourages directly observed therapy (DOT) by a healthcare agent because poor medication compliance is associated with poor outcomes. This may be difficult in low-income communities where travel on public transportation or into crowded slums poses risk of COVID-19 exposure to healthcare workers. Finally, we see our MDR-TB patients monthly in the clinic to provide medications and adherence counseling during outpatient therapy, but patients may be reluctant to visit the clinic during the COVID-19 pandemic. We recommend using health agents in the same community as patients to perform DOT. We have increased telephone communication with our MDR-TB patients, performing COVID symptom screening before clinic visits, and maintain frequent virtual contact to ensure continued engagement in care. We changed our follow-up schedule to accommodate longer-than-monthly intervals between visits to reduce the risk of nosocomial COVID-19. When economically feasible, MDR-TB patients should be provided with phone minutes to reduce the cost of frequent clinic calls.

### Potential increased risk of drug interactions.

The WHO recommends MDR-TB treatment regimens to include bedaquiline, linezolid, and fluoroquinolone with addition of clofazimine or cycloserine to achieve at least four effective drugs. These medications carry potential adverse side effects that may be exacerbated by symptoms associated with COVID-19. Bedaquiline, fluoroquinolones, and clofazimine can all cause QTc prolongation.^4^ SARS-CoV-2 infection is associated with cardiac involvement and myocardial inflammation, although the specific impact of this on patient outcomes is unknown.^9^ It is possible that the use of these antituberculous drugs in COVID-19 patients may induce more severe cardiac manifestations. Patients with COVID-19 can present with elevated liver function enzymes which may prevent the use of bedaquiline, a drug known for being hepatotoxic.^4^ The use of linezolid, another core drug in MDR-TB regimens, is associated with bone marrow toxicity. A unique sign of COVID-19 is the presence of lymphopenias, which we saw in our MDR-TB patient in Haiti and which 90% of patients in a New York City cohort demonstrated.^8^ Management of MDR-TB in people living with HIV is complicated by higher rates of drug toxicities that may be exacerbated in the setting of COVID-19 coinfection.^4^ The use of steroids in severe COVID-19 decreases mortality^10^ and may serve a role in reducing drug interactions in coinfected patients although more research is needed. To ensure that patients with both MDR-TB and COVID-19 are effectively managed, increased laboratory monitoring for lymphopenia, transaminitis, and arrhythmias is warranted. Multidrug-resistant TB medications may need to be held if such abnormalities occur.

### Infection control.

SARS-CoV-2 is transmitted via droplet and likely via airborne transmission as well.^11^ This has serious implications for many MDR-TB hospitals in resource-limited countries, such as Haiti, where respiratory isolation rooms do not exist. Nosocomial spread of COVID-19 among TB patients has already been documented^12^ and highlights the vulnerability of MDR-TB hospitals to this pandemic. Patients with MDR-TB must be screened for COVID-19 before hospitalization to prevent nosocomial transmission among MDR-TB patients and the healthcare workers caring for them. In addition, patients’ families should also be screened for both diseases. National ministries of health should consider transitioning to broader outpatient treatment of stable MDR-TB patients. With all-oral treatment regimens, the need for routine hospitalization of MDR-TB patients is decreasing. Care should be refocused to outpatient settings to prevent COVID-19 among hospitalized MDR-TB patients.

The COVID-19 pandemic may adversely affect the care of patients with MDR-TB. It is imperative to design triage pathways to detect MDR-TB, to monitor coinfected patients for rapid decline and drug interactions, and to ensure both MDR-TB patients and providers are protected from nosocomial transmission of COVID-19. Without preparation for these twin pandemics, we risk setting back MDR-TB care years if not decades.
